# From Fundamental Biointeractions to Advanced Clinical Therapies in Dentistry Using Biomaterials and Biological Components: An Editorial

**DOI:** 10.3390/jfb17060261

**Published:** 2026-05-28

**Authors:** Gustavo Vicentis Oliveira Fernandes

**Affiliations:** 1 Missouri School of Dentistry and Oral Health, A.T. Still University, St. Louis, MO 63104, USA; gustfernandes@gmail.com; 2 GF10 Foundation, St. Louis, MO 63104, USA

## 1. Introduction

The field of dental biomaterials has undergone a monumental shift, evolving from simple inert fillers to complex, bio-instructive systems capable of orchestrating tissue regeneration. This Special Issue, titled “Dental Biomaterials”, explores this journey, framing the progress of biomaterials within the context of bioengineering and clinical application. The rapid advancement of materials for grafting procedures, reconstructions, and prosthetic applications has outpaced many practitioners’ fundamental biological understanding, creating a gap between technical capability and biological rationale. This editorial synthesizes high-impact research on molecular signaling, hard and soft tissue substitutes, and updated microbiological paradigms. The recent literature demonstrates the efficacy of nanostructured bone grafts, such as hydroxyapatite (HA), and recombinant proteins (rhBMPs) in enhancing osseointegration, the clinical reliability of short/extra-short implants, and the regenerative potential of autologous platelet concentrates (APC) in the healing process. Furthermore, the role of cellular dynamics (ROS, LMW-PTP) and the macrophage-driven immune response are identified as critical factors for success. While the evolution of dental technology offers unprecedented opportunities, clinical success remains tethered to a deep comprehension of biological fundamentals. This editorial argues that navigating the “biomaterials’ world” requires more than just technical skill; it demands a return to biological literacy and knowledge of basic and advanced concepts to ensure long-term, predictable outcomes for patients.

## 2. The Technical—Biological Paradox

The progress in biomaterials applied in dental sciences, intrinsically linked to the evolution of bioengineering, has enabled a radical transformation in the materials used for bone and soft tissue grafts, implants, and prosthetic reconstructions. We are currently in an era where “bio-intelligence” is integrated into the biomaterials we use daily. However, as we embark on this journey “traveling through the biomaterials world”, we must confront a disturbing paradox in modern dentistry.

There is an undeniable fervor among dental professionals to adopt advanced procedures—be it guided implant surgery, the use of growth factors (GFs), or the application of complex monolithic ceramics. Yet, this enthusiasm for the “new” and “technological” is frequently accompanied by a significant lack of basic understanding regarding the biological foundations that dictate the success or failure of these materials. Many clinicians seek to master the “how” of a procedure while neglecting the “why” of the tissue response.

Choosing a biomaterial should not be a decision based on marketing or simplified clinical protocols; it must be an exercise in clinical reasoning rooted in cell biology, immunology, and biomechanics. Understanding advances in biomaterials enables appropriate applications and effective strategies to improve dental treatment outcomes.

This editorial aims to address this gap, providing a comprehensive overview of how biomaterials have evolved from passive structural replacements to active participants in tissue regeneration.

## 3. The Foundation: Molecular Signaling and the Cellular Interface

The “travel” begins at the subcellular level. A recurring criticism of contemporary clinical practice is the disregard for the initial events of the host–biomaterial interface. Before a clinician observes “osseointegration” on a radiograph, a complex series of molecular interactions must occur.

Early research into osteoblast adhesion dynamics revealed that cellular anchoring is not a static event but a highly regulated signaling process. The roles of Reactive Oxygen Species (ROS) and Low Molecular Weight Protein Tyrosine Phosphatase (LMW-PTP) have been highlighted as potential pathways for controlling osteoblast behavior. This suggests that the surface of a dental implant or a bone graft is not merely a physical scaffold but a chemical messenger that can trigger or inhibit bone formation.

Furthermore, we must reconsider the role of the immune system in the success of biomaterials. For years, the “foreign body reaction” was viewed negatively. However, modern perspectives emphasize that macrophages are central orchestrators of bone remodeling and osteogenesis. The transition from a pro-inflammatory (M1) to a pro-healing (M2) macrophage phenotype is the biological pivot upon which regenerative success turns. Clinicians who overlook this immunological component may struggle to explain why technically perfect graft procedures occasionally fail due to “unexplained” inflammation.

## 4. “Hard” Biomaterials: Navigating Bone Regeneration and Osseointegration

The evolution of “hard” biomaterials has moved toward biomimetics (materials that mimic the physical and chemical properties of natural bone). This section of our journey explores how we can manipulate the bone bed to accept and integrate various substitutes.

### 4.1. Advanced Bone Substitutes and Bioactive Enhancements

The development of nanostructured biomaterials represents a significant leap forward. Recent advancements emphasize the dual function of implant surfaces: supporting bone integration while actively limiting bacterial colonization. For instance, Martín-Santana et al. demonstrated that zinc-doped biomimetic calcium phosphate (CaP-Zn) coatings on surface-activated Ti6Al4V implants provide a multifunctional interface. Their work confirms that these biomimetic depositions significantly enhance bioactivity and osteoconductivity while introducing essential antimicrobial properties [1].

Similarly, addressing the critical clinical reality that bacterial infection is a primary driver of implant failure, Alenezi’s comprehensive systematic review and meta-analysis highlight the efficacy of modifying metallic implants with silver (Ag) coatings. The findings confirm that these targeted coatings not only boost antibacterial performance but also actively promote bone formation and osseointegration in animal models [2].

A comparison of biodegradable and ceramic hydroxyapatite (HA)-based grafts using synchrotron analysis and scanning electron microscopy (SEM) has shown that the synthesis process and resulting nanomorphology directly influence the biological response. This is further enhanced by the association of bone morphogenetic proteins (BMPs). For instance, recombinant human BMP-7 (rhBMP-7) associated with nanometric HA coatings on titanium surfaces has been shown to significantly improve osseointegration in preclinical models. Furthermore, the use of rhBMP-2 has been clinically validated to reduce volumetric contraction in post-extraction sockets (humans), a crucial consideration for ridge preservation [3].

In clinical practice, the choice among autologous, allogeneic, xenogeneic, or synthetic grafts remains a daily decision. A study comparing four bone grafts with expanded polytetrafluoroethylene (e-PTFE) membranes in critical-size defects in rats emphasizes that histomorphometric outcomes are highly dependent on the graft’s ability to maintain space and support cellular infiltration.

### 4.2. Pharmacological Interactions in Bone Healing

A critical area often overlooked by technically focused clinicians is the systemic influence of medications on biomaterial performance. The interaction between bisphosphonates (such as Zoledronate and Clodronate) and nanostructured HA grafts has been studied to foster better clinical reasoning. Understanding how these systemic modifiers affect local bone repair is essential for treating the modern, medically complex patient. We cannot treat the mouth as an isolated environment; it is a biological system influenced by systemic pharmacology and epigenetics.

### 4.3. The Evolution of Dental Implants: Materials and Dimensions

The “travel” through implantology reveals a shift toward microstructural optimization and precise biomechanical engineering. While commercially pure (CP) titanium is a staple due to its high biocompatibility, its relatively low strength can limit its use in highly loaded applications. Hlinka et al. evaluated ultrafine-grained (UFG) titanium, demonstrating that reducing the grain size and applying electrochemical surface modifications drastically improve the biological response, corrosion resistance, and overall technological properties of the implants [4].

Beyond material composition, the implant’s macrogeometry plays a profound role in tissue response. Gehrke et al. conducted an extensive in vitro and in vivo study on implant macrogeometry, revealing that controlling cortical bone compression during insertion is crucial. Their findings show that minimizing excessive compression yields a more favorable biological reaction and accelerates peri-implant bone healing, which is vital for long-term primary stability [5].

Furthermore, clinical success is tightly linked to patient-specific biomechanics. Abe et al. provided a retrospective analysis of female patients, demonstrating that the magnitude of occlusal force and masticatory performance strongly influence both patients’ selection of implant treatment for a missing mandibular second molar and the restoration’s long-term success [6].

In addition, the debate between titanium and zirconia remains a focal point. Meta-analyses of preclinical studies indicate that both materials achieve high levels of histological osseointegration, though zirconia offers specific advantages in soft tissue response and esthetics. Clinically, monolithic CAD/CAM zirconia restorations have proven to be robust and successful in tooth-supported applications.

Perhaps the most significant “technology applied to dentistry” in recent years is the refinement of implant dimensions. To avoid complex and morbidity-prone grafting procedures, the use of short and extra-short implants has gained traction. Robust evidence now supports the view that extra-short implants (≤6 mm) perform comparably to longer implants in terms of survival while significantly reducing the need for bone augmentation. Long-term retrospective studies with follow-ups exceeding 74 months have further confirmed the reliability of short implants (>6 mm and ≤8.5 mm) in the posterior segment. This evolution allows for a more patient-centric approach, prioritizing biological conservation.

## 5. Restorative Biomaterials: Interfaces and Environmental Resistance

The biological success of an implant or graft is intrinsically linked to its restorative counterpart. The durability and integration of modern prosthetics require a deep understanding of material interfaces and their behavior over time.

Şentürk et al. comparatively evaluated the shear bond strength (SBS) between monolithic zirconia (MZ) and various core build-up materials. The in vitro study provides essential guidelines for clinicians, proving that the choice of core material significantly impacts the mechanical retention and stability of highly esthetic zirconia reconstructions [7]. Moreover, these restorative materials are constantly challenged by the oral environment. Degirmenci et al. highlighted this by assessing CAD/CAM resin nanoceramics and demonstrating that aging factors such as thermocycling and ultraviolet (UV) exposure significantly alter surface characteristics and reduce repair bond strength over time [8].

Even in the transitional phase of treatment, provisional materials require bio-intelligence. Nazemi Salman et al. introduced a nanoclay-infused Cavit temporary filling, establishing its superior antibacterial characteristics. This innovation is crucial for sealing access cavities and protecting the internal environment during delayed final crown placements in pediatric and endodontic procedures [9].

## 6. Soft Tissue Biomaterials: From Hemostasis to Regeneration

Our journey continues into the realm of “soft” biomaterials, which are crucial for protecting the bone–implant interface and achieving esthetic harmony.

### 6.1. Autologous Platelet Concentrates (APC)

The shift from first-generation platelet-rich plasma (PRP) to second- and third-generation platelet concentrates—platelet-rich fibrin (PRF)—represents one of the most successful integrations of bioengineering into daily practice. These materials act as reservoirs for GFs, facilitating tissue repair and regeneration. Recent in vitro models have deeply expanded our understanding of these dynamics. Kirilova et al. evaluated the effects of various biomaterials derived from autologous blood on human dental pulp stem cells (hDPSCs). Their study confirmed that these advanced platelet concentrates significantly enhance stem cell migration, acting as bioactive scaffolds that recruit progenitor cells to the site of injury, thereby accelerating soft tissue and pulp regeneration [10].

Systematic reviews have highlighted the efficacy of these concentrates in many dental procedures, including the surgical treatment of gingival recessions. Specifically, the use of L-PRF in combination with coronally advanced flaps (CAFs) has shown acceptable results in treating Type 1 gingival recessions. Beyond periodontal surgery, liquid PRF has been used to coat implant surfaces to enhance the early stages of osseointegration through bioactive “bio-functionalization” of the titanium surface. This is a prime example of how simple biological technology can be applied to enhance mechanical systems.

### 6.2. Ridge Preservation and Wound Healing

The management of extraction sockets has also evolved to include soft materials, bone grafts, and hemostatic biomaterials. Assessment of collagen membranes and sponges in alveolar sockets has shown their prospective clinical value in stabilizing the initial clot and facilitating subsequent healing. Additionally, the use of autologous demineralized dentin matrix (DDM) for ridge preservation has emerged as a promising systematic alternative, utilizing the patient’s own tooth structure as a bioactive bone substitute.

## 7. Rethinking Failure: Microbiology, Peri-Implant Condition, and New Device for Treatment

No journey through the world of biomaterials is complete without acknowledging the threats to success. The lack of basic understanding regarding the oral microbiome is a major contributor to the rise in peri-implant diseases.

Modern research suggests we must move beyond the classical Socransky complexes. New bacterial clusters, proposed as the GF-MoR complexes, provide a summarized framework for understanding the polymicrobial nature of periodontal and peri-implant diseases [11]. Furthermore, the molecular pathways involving sclerostin and its inhibition (antisclerostin) are being explored to improve the bone remodeling environment around dental implants.

As we revisit peri-implant diseases, it is clear that we must rethink the future of compromised dental implants. The technology of tomorrow will not just involve better materials but better ways to manage the host–biofilm–biomaterial triad, utilizing scientifically validated protocols for surface refinement and re-osseointegration to salvage failing implants. Advancing the therapeutic frontier of peri-implantitis, the novel iMPACT Tool and Quadrant Protocol provide a scientifically validated pathway for implant surface refinement and successful re-osseointegration, effectively bridging high-resolution SEM/EDS analysis with long-term clinical predictability.

## 8. Conclusions: The Compass for the Journey

Traveling through the world of biomaterials is a journey of immense potential. The evolution from basic plates and screws to nanostructured, bioactive, and GF-enhanced systems has redefined what is possible in dental rehabilitation. However, the recurring point is to call readers to “Biological Responsibility”.

The modern professional must resist the urge to be a mere technician. To truly serve patients and improve treatment outcomes, one must master the biological principles that govern these materials. Technology applied to the dental area—be it CAD/CAM, short/extra-short implants, new devices, or APC—is only a tool. The true driver of success is the clinician’s ability to apply these tools within a framework of scientific reasoning. It is our hope that the diverse and rigorous research presented here serves as a compass for clinicians and researchers alike, guiding them toward a more integrated, biologically sound future in dental biomaterials.

## References

[1] Martín-Santana Y., González-Carranza Y., Díaz-Tato L., Juárez-Hernández A., García-Sánchez E.O., De La Garza-Ramos M.A., Rodríguez-Castellanos E.A., Hernández-Rodríguez M.A.L. (2026). Biomimetic Deposition of Zn-Doped Calcium Phosphate Coatings on Surface-Activated Ti6Al4V for Multifunctional Implant Interfaces. J. Funct. Biomater..

[2] Alenezi A. (2025). Effects of Implant Silver Coatings on Bone Formation in Animal Models: A Systematic Review and Meta-Analysis. J. Funct. Biomater..

[3] Araujo L.M.L., Prado W.M., Zenóbio E.G., Faverani L.P., de Souza J.G., Fernandes G.V.O., Shibli J.A. (2025). Effect of recombinant human Bone Morphogenetic Protein-2 (rhBMP-2) on the Volumetric Contraction of Post-Extraction Sockets: A Double-blinded Randomized Controlled Clinical Trial. Quintessence Int..

[4] Hlinka J., Cvejn D., Dluhos L., Babuska V., Cabanova K., Dvorakova J., Volodarskaja A., Valiev R.Z., Faisal N.H., Vodarek V. (2025). Grain Size and Electrochemical Surface Modification Effects on Corrosion, Biological, and Technological Properties of CP Titanium Implants. J. Funct. Biomater..

[5] Gehrke S.A., Aramburú Junior J., Treichel T.L.E., Scarano A., Mello B.F., Formiga M.d.C., Tari S.R., Coura G., Fernandes G.V.O. (2026). Influence of Reduced Cortical Bone Compression by Implant Macrogeometry on Peri-Implant Bone Healing: An In Vitro and In Vivo Experimental Study. J. Funct. Biomater..

[6] Abe T., Munakata M., Yokoi T., Yamaguchi K., Sato D., Baba K. (2025). Characteristics of Occlusal Force and Masticatory Performance in Female Patients Who Selected Implant Treatment for a Missing Mandibular Second Molar: A Retrospective Study. J. Funct. Biomater..

[7] Şentürk A., Akat B., Gönüldaş F., Halaçlı O.A., Kartal S., Kılıçarslan M.A. (2025). Comparative Evaluation of Shear Bond Strength Between Monolithic Zirconia and Three Different Core Build-Up Materials. J. Funct. Biomater..

[8] Degirmenci B.U., Degirmenci A., Seyfioglu Polat Z. (2025). The Influence of Thermocycling and Ultraviolet Aging on Surface Characteristics and the Repair Bond Strength of CAD/CAM Resin Nanoceramics. J. Funct. Biomater..

[9] Nazemi Salman B., Notash A., Ramazani A., Niaz S., Naeimi S.M., Darvish S., Luchian I. (2025). Antibacterial Characteristics of Nanoclay-Infused Cavit Temporary Filling Material: In Vitro Study. J. Funct. Biomater..

[10] Kirilova J., Vladova R., Petrova V., Yantcheva S., Deliverska E., Ishkitiev N. (2025). Influence of Different Biomaterials Extracted from Autologous Blood on the Cell Migration of Stem Cells from Dental Pulp. J. Funct. Biomater..

[11] Fernandes G.V.O., Mosley G.A., Ross W., Dagher A., Martins B.G.S., Fernandes J.C.H. (2024). Revisiting Socransky’s Complexes: A Review Suggesting Updated New Bacterial Clusters (GF-MoR Complexes) for Periodontal and Peri-Implant Diseases and Conditions. Microorganisms.

